# Electricity as an Anæsthetic

**Published:** 1859-09

**Authors:** 


ELECTRICITY AS AN ANÆSTHETIC.—A great deal has recently been written in regard to the application of electricity to the extraction of teeth, but it does not seem to realize the hopes of its introducers, and has by no means proved a success as an anæsthetic. The Pennsylvania Association of Surgeon-Dentists and the St. Louis Dental Society have each appointed committees to test its value, and their reports confirm each other, that in a very few cases the operation was said to be entirely painless; while in the great majority of instances the pain was slightly relieved, or not at all, and occasionally it was increased. A committee of the College of Dentists of London, in a recent report, stated that the results were decidedly negative, and that in no instance was local anæsthesia produced. In a scientific point of view, it, therefore, condemned the employment of the electrical current as a means of diminishing pain in dental operations.

